# A Survey on Deep Reinforcement Learning Algorithms for Robotic Manipulation

**DOI:** 10.3390/s23073762

**Published:** 2023-04-05

**Authors:** Dong Han, Beni Mulyana, Vladimir Stankovic, Samuel Cheng

**Affiliations:** 1 School of Electrical and Computer Engineering, University of Oklahoma, Norman, OK 73019, USA; 2 Department of Electronic and Electrical Engineering, University of Straclyde, Glasglow G1 1XW, UK

**Keywords:** reinforcement learning, robotic manipulation, graph neural network

## Abstract

Robotic manipulation challenges, such as grasping and object manipulation, have been tackled successfully with the help of deep reinforcement learning systems. We give an overview of the recent advances in deep reinforcement learning algorithms for robotic manipulation tasks in this review. We begin by outlining the fundamental ideas of reinforcement learning and the parts of a reinforcement learning system. The many deep reinforcement learning algorithms, such as value-based methods, policy-based methods, and actor–critic approaches, that have been suggested for robotic manipulation tasks are then covered. We also examine the numerous issues that have arisen when applying these algorithms to robotics tasks, as well as the various solutions that have been put forth to deal with these issues. Finally, we highlight several unsolved research issues and talk about possible future directions for the subject.

## 1. Introduction

**Figure 1:**
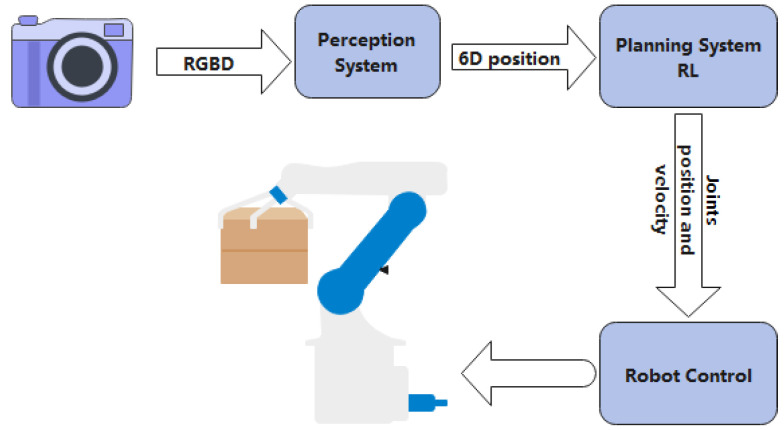
Classic robotic manipulation workflow.

Industry 4.0’s embrace of artificial intelligence (AI) has drastically changed the industrial sector by allowing machines to operate autonomously, increasing productivity, lowering costs, and improving product quality [1]. In order to make decisions quickly and efficiently during the manufacturing process, artificial intelligence (AI) technology analyzes enormous amounts of data produced by machines and processes. In the next five years, its total market value is expected to triple [2]. Robotics is one of the markets that is anticipated to expand at the fastest rates. With the concept of robot manipulation proposed in 1962 [3], the idea of a robot is to mimic human behavior and tackle complex tasks. A branch of robotics called robotic manipulation is focused on developing robots that can manipulate items in their environment. Robotic manipulation seeks to develop robotic systems that can carry out a variety of activities that call for the manipulation of things, such as putting together goods in a factory, picking and placing items in a warehouse, or performing surgery in a hospital. Robotic manipulation systems typically consist of a robot arm, a gripper or end-effector, and a control system that coordinates the movement of the robot arm and gripper. The gripper is in charge of grabbing and manipulating items, while the robot arm is in charge of transferring them to the target area in the environment. Figure 1 shows a classic robotic manipulation workflow. Moreover, Matt Mason provided a thorough and in-depth description of manipulation in the introduction of his 2018 review paper [4]. Robotic manipulation is used in various fields such as manufacturing, agriculture, healthcare, logistics, space exploration, education, and research. It entails using robots to carry out activities including assembling, planting, harvesting, operating, managing goods, and performing experiments. In a variety of tasks, robotic manipulation can boost productivity, cut human costs, increase accuracy, and enhance safety. In the upcoming years, its application is anticipated to increase across a range of industries. In the manufacturing sector, robots are used for tasks including component assembly, welding, painting, and packaging. They have the ability to work carefully and diligently, increasing productivity and decreasing costs. In the healthcare sector, robots may assist with tasks including surgery, rehabilitation, and geriatric care. They can support healthcare personnel by letting them focus on more challenging tasks while the robot handles the basic tasks. In space exploration, robots are used to complete activities including sample gathering, structure construction, and equipment repair. They can operate in environments that are too dangerous or challenging for people, such as deep space or the ocean floor. However, the interaction between robotic manipulator arms and objects designed for humans remains a challenge. Up to now, no robots can easily achieve intelligent operations such as handwashing dishes, peeling a pineapple, or rearranging furniture.

A subfield of artificial intelligence called deep reinforcement learning combines reinforcement learning and deep learning, a technique for training artificial neural networks [5]. Robots are programmed with a set of rules and instructions that specify how they should interact with items in their surroundings in conventional methods of robotic manipulation. This approach is effective for simple activities, but it becomes more challenging as the difficulty of the tasks increases. Robots may manipulate objects in their surroundings using a process called deep reinforcement learning, which allows them to make mistakes and learn from them. Deep reinforcement learning provides a more flexible and adaptable method, allowing robots to learn from experience and change their behavior [6,7]. For instance, the robot receives positive reinforcement if it successfully picks up and moves an object to the desired location. If it drops the object or fails to transfer it to the desired location, a negative reward is provided. Because it has the capacity to correlate some activities with good results and other actions with undesirable outcomes, over time, the robot develops a strategy for accomplishing the task at hand [8]. Robotic manipulation using deep reinforcement learning has the potential to change a variety of industries, including healthcare and manufacturing. Allowing robots to learn from experience and adjust to changing situations enables them to perform tasks that are too difficult or dangerous for humans to complete. As research in this area advances, we can expect to see more capable and advanced robots that can manipulate objects more precisely and effectively.

RL has been successfully applied to a wide range of problems in various fields, including game-playing, recommendation systems, and finance. One successful use of RL is in the game of Go [9], where DeepMind’s AlphaGo program defeated the world champion Lee Sedol in 2016 to attain unparalleled success. In addition to its application in finance to improve trading tactics and portfolio management, RL has been used to create recommendation systems that can learn to give individualized suggestions depending on user behavior [10]. Moreover, RL has been used for complicated issues in which conventional rule-based systems or supervised learning approaches fall short, including those in the domains of natural language processing [11], drug discovery [12], and autonomous driving [13], among others. Creating algorithms that can precisely detect and distinguish objects in images or video streams is one of the primary issues in computer vision. By giving agents performance-based feedback in the form of rewards or penalties, RL can be utilized to teach agents to recognize objects [6]. As RL algorithms are capable of learning from experience and adapting to shifting settings, they provide a promising solution to a wide range of difficult issues in several industries.

In this survey, we will examine the key concepts and algorithms that have been developed for DRL in the context of robotic manipulation. This will include a review of techniques for reward engineering, such as imitation learning and curriculum learning, as well as approaches to hierarchical reinforcement learning. We will also discuss the various network architectures that have been used in DRL for robotic manipulation and the challenges associated with transferring learned policies from simulation to the real world. Finally, we will review both value-based and policy-based DRL algorithms and their relative strengths and limitations for robotic manipulation tasks. The contributions of the paper are:

- A tutorial of the current RL algorithms and reward engineering methods used in robotic manipulation.
- An analysis of the current status and application of RL in robotic manipulation in the past seven years.
- An overview of the current main trends and directions in the use of RL in robotic manipulation tasks.

The rest of the paper is organized as follows. The methodology used to find and choose relevant publications is described in Section 2. In Section 3, we introduce the key RL concepts and state-of-the-art algorithms. Next, Section 4 continues by describing the learning methods for DRL. In Section 5, we discuss the current neural network architectures in RL. In Section 6, we take a deep dive into the applications and implementations of robotic manipulation. Then, we describe the existing challenges and future directions with respect to previous work. The final paragraph of Section 7 provides a summary of the knowledge obtained.

## 2. Search Methodology

Since RL is more adaptable in highly dynamic and unstructured environments than more traditional or other AI control approaches, there has been a recent increase in interest in using RL to operate robotic manipulators [14]. These techniques have demonstrated impressive results in enabling robots to learn complex tasks and operate in dynamic environments. Moreover, the growing interest in robotic manipulation in reinforcement learning has been stimulated by the expanding availability of affordable and efficient robotic hardware, as well as the rising demand for automation across a variety of industries. Applications of this technology include manufacturing, logistics, healthcare, and home automation, among others. So, the purpose of this review is to present an overview of the major works using RL in robotic manipulation tasks and to analyze the future directions of this topic. In order to achieve this goal, a thorough review of the literature is conducted, and the content of more than 150 articles in relevant fields is searched and reviewed.

Given the enormous amount of literature on the subject, looking for papers on reinforcement learning for robotic manipulation can be a difficult task. Start by identifying the relevant keywords for the search, such as “reinforcement learning”, “robotic manipulation”, “manipulation tasks”, “control policies”, “deep learning”, etc. These keywords will help narrow down the search to the most relevant papers. Use a specialized search engine such as Google Scholar, IEEE Xplore, or ArXiv to search for papers related to reinforcement learning for robotic manipulation. These search engines allow for filtering the results from 2015 to 2022. This period’s start was chosen because of the release of the RL and the deep neural network using the AlphaGo program [15]. This innovation has made a significant contribution to the rapid growth of RL.

Meanwhile, studies that were not appropriate for the scope of this review had to be excluded even though they were relevant to the field of RL. Studies that are outdated or do not contribute to the current state of the field should be excluded. Additionally, the authors decided to exclude papers that are poorly written or that have significant methodological flaws. An overview of the specified search criteria can be found in Table 1.

## 3. Key Concepts and Algorithms of Reinforcement Learning

**Figure 2:**
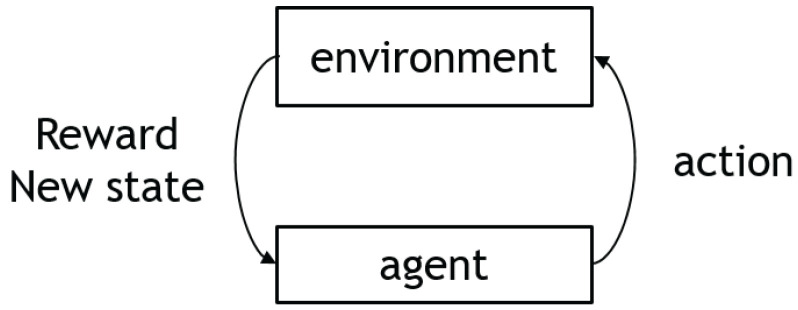
Block diagram of typical RL.

Reinforcement learning is a strategy that encourages an agent to take action and interact with an environment in order to maximize the total rewards. The agent–environment interaction process is shown in Figure 2. The agent takes action and receives feedback from the environment in the form of rewards or punishments. The agent uses this feedback to adjust its behavior and improve its performance over time.

An autonomous agent observes the state s(t) at a time step *t* and then interacts with the environment using an action a(t), reaching the next state s(t+1) in the process. Once a new state has been achieved, the agent receives a reward correlated with that state r(t+1). The agent’s goal is to find an optimal policy, i.e., the optimal action in any given state. Unlike other types of machine learning—such as supervised and unsupervised learning—reinforcement learning can only be thought about sequentially in terms of state-action pairs that appear one after the other.

RL assesses actions by the outcomes, i.e., the states, they achieve. It is goal-oriented and seeks to learn sequences of actions that will lead an agent to accomplish its goal or optimize its objective function. An example of the RL objective function is:

(1) $$\sum_{t=0}^{t=\infty }\gamma^{t}r\left(s\left(t\right),a\left(t\right)\right)$$

This objective function measures all of the rewards that we will receive from running through the states while exponentially increasing the weight γ.

Two important concepts of RL are Monte Carlo learning, which is a naive idea in which the agent interacts with the environment and learns about the states and rewards, and temporal difference (TD) learning, i.e., updating the value at every time step rather than being required to wait to update the values until the end of the episode.

**Figure 3:**
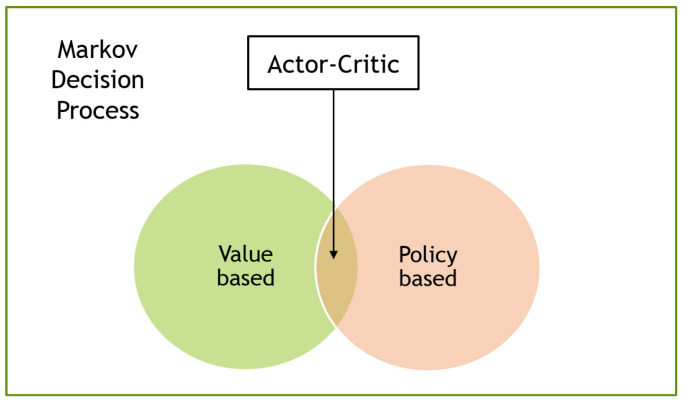
Types of RL algorithms.

Although it is difficult to make a standardized classification of RL algorithms due to their wide modularity, many current studies tend to divide them into value-based, policy-based, and actor–critic algorithms (see Figure 3).

### 3.1. Markov Decision Process and RL

A Markov decision process (MDP) is defined as a tuple 〈S,A,r,T,γ〉 where *S* stands for a set of states; *A* stands for actions; *r*, S×A→R denotes the function specifying a reward of taking an action in a state; T:S×A×S→R denotes the state transition function; and γ stands for the discount factor implying that a reward obtained in the future is worth a smaller amount than an immediate reward. Solving an MDP involves finding a policy that determines the optimal action for each state, with the goal of maximizing the long-term discounted expected reward. This policy should be optimal with respect to the MDP’s reward structure and discount factor.

### 3.2. Value-Based RL

#### 3.2.1. Q-Learning

Q-learning [16] is a value-based TD method of reinforcement learning that uses Q-values (also called state-action values) to iteratively develop the actions of the learning agent. Q-learning learns the Bellman action-value function Q(s,a), which estimates how good it is to take an action at a given state.

(2) $$Q\left(s,a\right)=r\left(s,a\right)+\gamma \max_{a}Q\left(s^{\prime },a\right).$$

The Bellman action-value equation describes how to calculate the Q-value for an action taken from a particular state, *s*. It is calculated as the sum of the immediate reward for the current state and the discounted optimal Q-value for the next state, denoted by γ. In Q-learning, the Q-value is updated using the following rule: The new Q-value is equal to the old Q-value plus the temporal difference error. This update can be framed as trying to minimize a loss function, such as the mean squared error loss:

(3) $$\;L\;={\left(r+\gamma \max_{a^{\prime }}Q\left(s^{\prime },a^{\prime };\theta^{\prime }\right)-Q\left(s,a;\theta \right)\right)}^{2}.$$

#### 3.2.2. SARSA

The SARSA [17] algorithm is a policy-based variant of the well-known Q-learning algorithm. Unlike Q-learning, which is an off-policy technique that learns the Q-value using a greedy approach, SARSA is an on-policy technique that uses the action taken by the current policy to learn the Q-value. To update Q-values, we use:

(4) $$Q\left(s,a\right)=Q\left(s,a\right)+\alpha \left[R+\gamma Q\left(s^{\prime },a^{\prime }\right)-Q\left(s,a\right)\right].$$

SARSA is a type of on-policy reinforcement learning algorithm, meaning that it learns the value of actions based on the policy that is currently being followed. In SARSA, the next action, $a^{\prime }$, is chosen using the same epsilon-greedy policy as the action that led to the current state, $s^{\prime }$. One advantage of this approach is that SARSA is able to learn a near-optimal policy while still allowing for some exploration. However, if the goal is to learn the optimal policy, it may be necessary to carefully tune the decay rate of the epsilon value in the epsilon-greedy action selection process. On the other hand, Q-learning is an off-policy algorithm that learns the optimal policy directly. While this can be more efficient in certain cases, it also has a higher per-sample variance and can be more difficult to converge when used with neural networks.

#### 3.2.3. Deep Q-Learning (DQN)

One problem with traditional Q-learning is that the size of the Q-table grows exponentially with the number of states and actions, making it impractical for many problems. To address this, deep Q-learning (DQN) was introduced by Mnih et al. [18], which uses a neural network to approximate the Q-values. As a universal function approximator, the neural network is able to capture the relationships between states and actions more efficiently than the Q-table.

**Figure 4:**
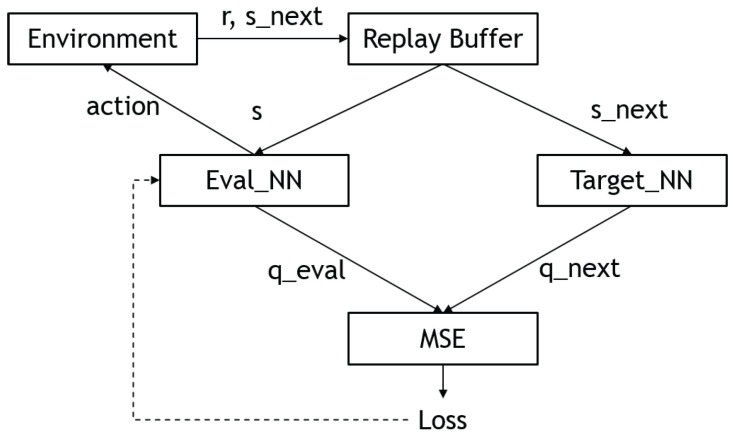
Flowchart of DQN.

However, one issue with using a neural network to learn the Q-values is that the update rule (Equation (4)) depends on the values produced by the network itself, which can make convergence difficult. To address this, the DQN algorithm introduces the use of a replay buffer and target networks. The replay buffer stores past interactions as a list of tuples, which can be sampled to update the value and policy networks. This allows the network to learn from individual tuples multiple times and reduces dependence on the current experience. The target networks are time-delayed copies of the policy and Q-networks, and their parameters are updated according to the following equations:

(5) $$\theta^{Q^{\prime }}\leftarrow \tau \theta^{Q}+\left(1-\tau \right)\theta^{Q^{\prime }}$$

(6) $$\theta^{\mu^{\prime }}\leftarrow \tau \theta^{\mu }+\left(1-\tau \right)\theta^{\mu^{\prime }}$$

where $\theta^{\mu^{\prime }}$ and $\theta^{Q^{\prime }}$ denote the parameters of the policy and Q-networks, respectively. Figure 4 shows the overall workflow of a deep Q-network.

#### 3.2.4. Double Deep Q-Learning (Double DQN)

One issue with the DQN algorithm is that it tends to overestimate the true rewards, leading to inflated Q-values. To address this, the double DQN algorithm [19] introduces a modification to the Bellman equation used in DQN. Instead of using the same equation, the action selection and action evaluation are decoupled in the following way:

(7) $$Q\left(s,a;\theta \right)=r+\gamma Q\left(s^{\prime },{\mathrm{argmax}}_{a^{\prime }}Q\left(s^{\prime },a^{\prime };\theta \right);\theta^{\prime }\right)$$

Here, the main neural network, θ, determines the best next action, $a^{\prime }$, while the target network is used to evaluate this action and compute its Q-value. This simple change has been shown to reduce overestimations and lead to better final policies.

#### 3.2.5. Dueling Deep Q-Learning (Dueling DQN)

The dueling DQN algorithm introduced by Wang et al. [20] seeks to improve upon traditional DQN by decomposing the Q-values into two separate components: the value function, V(s), and the advantage function, A(s,a). The value function represents the expected reward for a given state, *s*, while the advantage function reflects the relative advantage of taking a particular action, *a*, compared to other actions. By combining these two functions, it is possible to compute the full Q-values for each state-action pair.

To implement this decomposition, the dueling DQN algorithm introduces a neural network with two separate output layers, one for the value function and one for the advantage function. These outputs are then combined to produce the final Q-values. This modification allows the network to learn more efficiently in situations where the exact values of individual actions are not as important, as it can focus on learning the value function for the state.

### 3.3. Policy-Based RL

Policy gradient (PG) methods are widely used reinforcement learning algorithms that are particularly well-suited to situations with continuous action spaces [21]. The goal of an RL agent using a PG method is to maximize the expected reward, $J\left(\pi_{\theta }\right)=\underset{\tau ∼\pi_{a}}{E}\left[R\left(\tau \right)\right]$, by adjusting the policy parameters, θ. A standard approach to finding the optimal policy is to use gradient ascent, in which the policy parameters are updated according to the following rule:

(8) $$\theta_{t+1}=\theta_{t}+\alpha \nabla J\left(\pi_{\theta_{t}}\right)$$

where α is the learning rate, and $\nabla J\left(\pi_{\theta }\right)$ is the policy gradient. This gradient can be further expanded and reformulated as:

(9) $$\nabla J\left(\pi_{\theta }\right)=\underset{\tau ∼\pi_{\theta }}{E}\left[\sum_{t=0}^{T}\nabla_{\theta }\log\pi_{\theta }\left(a_{t}|s_{t}\right)R\left(\tau \right)\right].$$

In PG methods, the policy function, which maps states to actions, is learned explicitly and actions are selected without using a value function.

#### 3.3.1. Vanilla Policy Gradient (VPG)

In RL, it is often more important to understand the relative advantage of a particular action, rather than its absolute effectiveness. The advantage function, $A_{\pi }\left(s,a\right)$, captures this idea by measuring how much easier it is to take a specific action, *a*, in a state *s*, compared to randomly selecting an action according to the policy, π, considering that the policy will be followed indefinitely thereafter. Mathematically, the advantage function is defined as:

(10) $$A_{\pi }\left(s,a\right)=Q_{\pi }\left(s,a\right)-V_{\pi }\left(s\right)$$

where $Q_{\pi }\left(s,a\right)$ is the action-value function, and $V_{\pi }\left(s\right)$ is the state-value function for the policy π. Using this definition, the vanilla policy gradient (VPG) algorithm [21] can be written as:

(11) $$\nabla J\left(\pi_{\theta }\right)=\underset{\tau ∼\pi_{\theta }}{E}\left[\sum_{t=0}^{T}\nabla_{\theta }\log\pi_{\theta }\left(a_{t}|s_{t}\right)A_{\pi }\left(s,a\right)\right]$$

**Figure 5:**
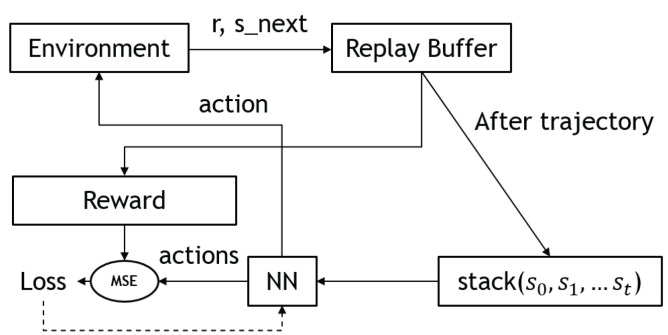
Flowchart of vanilla policy gradient.

Figure 5 describes the general workflow of the vanilla policy gradient.

#### 3.3.2. Trust Region Policy Optimization (TRPO)

In policy gradient methods, we aim to optimize a policy objective function, such as the expected cumulative reward, using gradient descent. These methods are well-suited for continuous and large state and action spaces but can be sensitive to the learning rate. A small learning rate may result in vanishing gradients, while a large learning rate may cause exploding gradients. Trust region policy optimization (TRPO) [22] was introduced as a solution to this problem, by constraining the optimization of a policy to a trust region. This region is defined as the area in which local approximations of the function are accurate, and the maximum step size is determined within it. The trust region is then iteratively expanded or shrunk based on how well the new approximation performs.

The policy update in TRPO is given by the following optimization problem, which uses the Kullback–Leibler (KL) divergence between the old and new policies as a measure of change:

(12) $$\nabla J\left(\pi_{\theta }\right)=\underset{\tau ∼\pi_{\theta }}{E}\left[\frac{\pi \theta \left(a_{t}|st\right)}{\pi \theta_{\mathrm{old}\;}\left(a_{t}|st\right)}\hat{A}t\right]$$

(13) $$\;\mathrm{subject}\;\mathrm{to}\;\underset{\tau ∼\pi \theta }{E}\left[\mathrm{KL}\left[\pi \theta_{\mathrm{old}\;}\left(\cdot |st\right),\pi \theta \left(\cdot |st\right)\right]\right]\leq \delta$$

where δ is the size of the trust region, and the KL divergence between the old and new policies must be less than δ. This optimization problem can be solved using the conjugate gradient method.

#### 3.3.3. Proximal Policy Optimization (PPO)

Proximal policy optimization (PPO) [23] is an algorithm that aims to address the overhead issue of TRPO by incorporating the constraint into the objective function as a penalty. Instead of adding a separate constraint, the KL divergence between the old and new policies is subtracted from the objective function and multiplied by a constant *C*. This allows for the use of simple stochastic gradient descent to optimize the function:

(14) $$\underset{\tau ∼\pi_{\theta }}{E}\left[\frac{\pi \theta \left(a_{t}|st\right)}{\pi \theta_{\mathrm{old}\;}\left(a_{t}|st\right)}\hat{A}t\right]-C\cdot \bar{\mathrm{KL}}\pi \theta_{\mathrm{old}\;}\left(\pi \theta \right)$$

One challenge of PPO is choosing the appropriate value for *C*. To address this, the algorithm updates *C* based on the magnitude of the KL divergence. If the KL divergence is too high, *C* is increased, and, if it is too low, *C* is decreased. This allows for effective optimization over the course of training.

### 3.4. Actor–Critic

**Figure 6:**
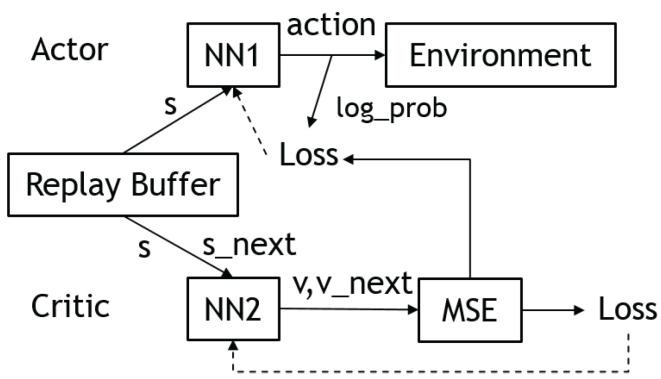
Flowchart of actor–critic.

Actor–critics [24] are RL algorithms that combine elements of both value-based and policy-based methods. In this approach, the actor, a policy network, proposes an action for a given state, while the critic, a value network, evaluates the action based on the state-action pair. The critic uses the Bellman equation to learn the Q-function, and the actor is updated based on the Q-function to train the policy. This allows the actor–critic approach to take advantage of the strengths of both value-based and policy-based methods. Figure 6 illustrates the network architecture of the actor–critic.

#### 3.4.1. Advantage Actor–Critic (A2C)

In the advantage actor–critic (A2C) algorithm [25], the critic is trained to estimate the advantage function instead of the Q-function. This allows the evaluation of an action to consider not only its success but also how much better it is compared to other actions. Using the advantage function can help reduce the high variance of policy networks and improve the stability of the model.

#### 3.4.2. Asynchronous Advantage Actor–Critic (A3C)

The asynchronous advantage actor–critic (A3C) algorithm [25], released by DeepMind in 2016, is a highly efficient and effective reinforcement learning algorithm that has outperformed other methods, such as DQN, on many tasks. A key feature of A3C is its asynchronous nature, which permits multiple independent agents (networks) with their own weights to interact with different copies of the environment in parallel, allowing for more efficient exploration of the state-action space. A3C has proven to be a robust and reliable method, achieving high scores on standard reinforcement learning tasks.

#### 3.4.3. Deep Deterministic Policy Gradient (DDPG)

Deep deterministic policy gradient (DDPG) [26] is a reinforcement learning technique that combines both deep Q-learning (DQN) [27] and deterministic policy gradients (DPG) [28]. DDPG is an actor–critic technique, it uses two neural networks: a deterministic policy network and a critic (Q) network. The policy network simply performs gradient ascent to solve Equation (4). Note that the critic parameters are frozen as constants. The critic network is updated similarly to Equation (3). Nevertheless, in DDPG, the updated Q-values are calculated by the Bellman Equation (2) with the target Q-network and target policy network. Then, we minimize the mean squared error loss between the original Q-value and the updated Q-value:

(15) $$\;L\;=\frac{1}{N}\sum_{i}{\left(Q_{old}-\left(r\left(s,a\right)+\gamma \max_{a}Q_{target}\left(s^{\prime },a\right)\right)\right)}^{2}$$

**Figure 7:**
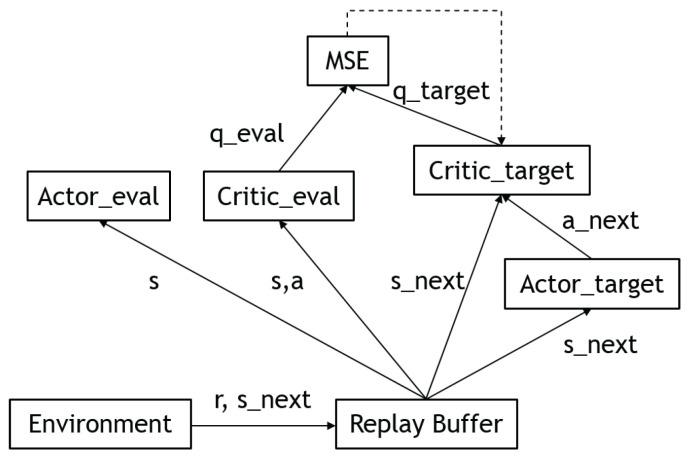
Flowchart of Deep Deterministic Policy Gradient.

Since the policy is deterministic, it suffered from inefficient exploration when the agent was to explore the environment. To improve the DDPG policy, the authors added Ornstein–Uhlenbeck noise [29] to the selected actions during training. However, more recent research implies that uncorrelated, zero-mean Gaussian noise is effective. Figure 7 shows the updated rule of the deep deterministic policy gradient.

#### 3.4.4. Twin Delayed Deep Deterministic Policy Gradients (TD3)

Twin delayed deep deterministic policy gradients (TD3) [30] are the successor to DDPG. Although DDPG is capable of providing excellent results, it has its drawbacks. Similar to many RL algorithms, training DDPG can be unstable and heavily reliant on finding the correct hyperparameters for the current task. This is caused by the algorithm continuously overestimating the Q-values of the critic (value) network. These estimation errors build up over time and can lead to the agent falling into a local optimum or experiencing catastrophic forgetting. TD3 addresses this issue by focusing on reducing the overestimation bias seen in previous algorithms.

TD3 has three main features that help to solve the aforementioned problems. Firstly, TD3 uses two critic networks instead of one, and it uses the smaller of the two Q-values as the targets in the Bellman error loss functions. This helps to reduce overestimation by ensuring that the Q-value targets are more conservative. Secondly, TD3 updates the policy (and target networks) less frequently than the Q-function, which is called twin delay. The paper suggests one policy update for every two Q-function updates. This helps to reduce over-fitting and improve the stability of the algorithm. Finally, TD3 adds noise to the target action to make it harder for the policy to exploit Q-function errors by smoothing out Q along changes in the action. This helps to improve the robustness of the algorithm and reduce its reliance on hyperparameter tuning.

#### 3.4.5. Soft Actor–Critic (SAC)

Developed jointly by UC Berkeley and Google [31], soft actor–critic (SAC) is a cutting-edge reinforcement learning algorithm. It employs a maximum entropy approach in which the goal is to determine the optimal policy that maximizes both the expected long-term reward and the long-term entropy [32]. The objective function for this maximum entropy RL is displayed below:

(16) $$J\left(\pi_{\theta }\right)=E_{\pi_{\theta }}\left[\sum_{t=0}^{T-1}\gamma^{t}R\left(s_{t},a_{t}\right)+\alpha H\left(\pi \left(.|s_{t}\right)\right)\right]$$

The maximum entropy reinforcement learning objective is used to encourage exploration. This is achieved by promoting policies that assign equal probabilities to actions that have similar or almost equal Q-values, which leads to higher entropy. By explicitly encouraging exploration in this way, SAC is able to effectively explore the state-action space and find optimal policies.

SAC makes use of three networks: a state value function *V* parameterized by ψ, a policy function parameterized by ϕ, and a final network that represents a soft state-action value function parameterized by θ. We train the state value network by minimizing the following error:

(17) $$E_{s_{t}∼D}\left[\frac{1}{2}{\left(V_{\psi }\left(s_{t}\right)-E_{a_{t}∼\pi_{\phi }}\left[Q_{\theta }\left(s_{t},a_{t}\right)-\log\pi_{\phi }\left(a_{t}|s_{t}\right)\right]\right)}^{2}\right]$$

The loss function implies that across all of the states that we sampled from our experience replay buffer, we need to decrease the squared difference between the prediction of our value network and the expected prediction of the Q-function minus the entropy of the policy function π. We train the soft Q-network by minimizing the following error:

(18) $$J_{Q}\left(\theta \right)=E_{\left(s_{t},a_{t}\right)∼D}\left[\frac{1}{2}{\left(Q_{\theta }\left(s_{t},a_{t}\right)-\hat{Q}\left(s_{t},a_{t}\right)\right)}^{2}\right]$$

where

(19) $$\hat{Q}\left(s_{t},a_{t}\right)=r\left(s_{t},a_{t}\right)+\gamma E_{s_{t+1}∼p}\left[V_{\bar{\psi }}\left(s_{t+1}\right)\right]$$

In simple words, training is performed by minimizing the squared difference between the predicted Q-value and the reward plus the discounted expectation of the state-value of the next state. Finally, policy network learning is based on:

(20) $$J_{\pi }\left(\phi \right)=E_{S_{t}∼D}\left[D_{\mathrm{KL}}\left(\pi_{\phi }\left(\cdot |s_{t}\right)∥\exp\left(Q_{\theta }\left(s_{t},\cdot \right)\right)\right)\right]$$

attempting to make the distribution of our policy function look more similar to the distribution of the exponentiation of our Q-function.

After the discussion of RL algorithms, there are two possible approaches for finding the optimal policy: on-policy and off-policy. These two terms are used to describe how, in a general sense, the agent learns and behaves during the training phase, as well as the two main ways that an agent can go about learning/behaving. Table 2 shows categories of the different RL algorithms.

While RL has proven to be a powerful approach to solving a wide range of problems, there are several different algorithms that have been developed to address different types of environments and learning objectives. In this context, it is important to understand the strengths and limitations of these algorithms in order to make informed decisions about which algorithm to use for a particular application. Table 3 describes the strengths and limitations of the different RL algorithms.

## 4. Reward Engineering

### 4.1. Imitation Learning

**Figure 8:**
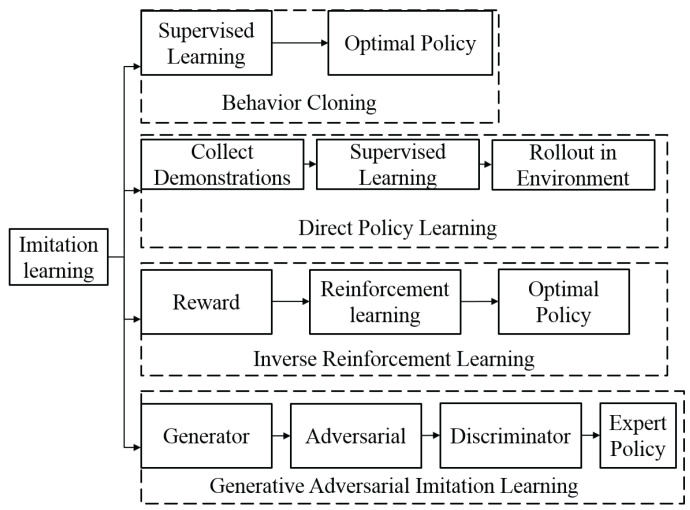
Classification of imitation learning [3].

In imitation learning, the goal is to learn a policy that can mimic the behavior of an expert. The expert’s behavior is represented as a set of demonstrations, which can be used to train the policy. The policy is typically learned by minimizing the distance between the expert’s behavior and the policy’s behavior, using a distance measure such as the KL divergence or the maximum mean discrepancy. The advantage of imitation learning is that it does not require a reward function to be specified, which can be difficult or infeasible in some cases. However, it may not generalize well to situations that are different from those encountered by the expert. The classification of imitation learning can be seen in Figure 8.

#### 4.1.1. Behavior Cloning

One popular algorithm for imitation learning is behavior cloning, where the goal is to learn a policy that can mimic the expert’s behavior. This can be achieved by collecting a dataset of expert demonstrations and using it to train a supervised learning algorithm to predict the expert’s actions given the current state. The learned policy can then be used to execute the desired behavior in the environment. Another approach is inverse reinforcement learning, where the goal is to learn the reward function that the expert is optimizing, and then use this reward function to train a policy using RL techniques. This allows the agent to not only mimic the expert’s behavior but also adapt and improve upon it. An important example of behavior cloning is ALVINN [33], a vehicle equipped with sensors that has learned to map the sensor inputs into steering angles and drive autonomously. Dean Pomerleau initiated this research in 1989, and it was also the first implementation of imitation learning in general.

#### 4.1.2. Direct Policy Learning

In direct policy learning (DPL), the goal is to learn a policy that can replicate the expert’s behavior as closely as possible. This is achieved through an iterative process where the policy is trained using supervised learning on a dataset of demonstrations provided by the expert. The trained policy is then implemented in the environment and evaluated through interactions with the expert. Any additional data collected during these interactions are then added to the training dataset, and the process is repeated until the policy converges to a satisfactory level of performance. RL-teacher [34] is a specific implementation of DPL that allows for the learning of novel behaviors without the need for a pre-defined reward function or the ability for the expert to demonstrate the desired behavior directly.

#### 4.1.3. Inverse Reinforcement Learning

Inverse reinforcement learning (IRL) [35] is a kind of imitation learning in which we are given a policy or a history of behavior from an agent and use reinforcement learning to try to discover a reward function that explains the behavior. IRL, similar to RL, is seen as an issue as well as a category of techniques. However, there are two issues with discovering a reward function that is best for observed behavior. First, for most observations of behavior, there are many fitting reward functions. Many degenerate solutions exist in the set of solutions, such as providing a reward of 0 to all states. Second, the IRL algorithms are based on the assumption that the observed behavior is ideal. This is similar to over-fitting in supervised learning.

IRL is a useful tool for understanding the motivations behind an agent’s behavior, but it can be difficult to apply it in practice due to the ambiguities mentioned above. In order to address these issues, researchers have proposed various techniques, such as maximum entropy IRL [36] and Bayesian IRL [37]. These approaches aim to overcome the ambiguities of IRL by incorporating additional assumptions or constraints, such as assuming that the reward function is smooth or that the agent is rational. Despite these efforts, IRL remains an active area of research and is not yet a widely used technique in practical applications.

#### 4.1.4. Generative Adversarial Imitation Learning (GAIL)

Generative adversarial imitation learning (GAIL) [38] combines IRL with generative adversarial networks (GAN). GAIL’s purpose is to train generators that behave similarly to given experts. Meanwhile, the discriminators may be used as reward functions for RL, which determines whether the actions match those of experts. GAIL can learn from a small number of expert trajectories. GAIL is capable of handling difficult issues and generalizes effectively to unknown scenarios. GAIL is not exactly IRL because GAIL learns the policy, rather than the reward function, directly from the data.

#### 4.1.5. Goal-Conditioned Imitation Learning (GCIL)

Goal-conditioned imitation learning (GCIL) [39] is a combination of the GAIL and hindsight experience replay (HER) [40] algorithms. GCIL utilizes the benefits of both algorithms, such as the ability of GAIL to quickly learn from a few demonstrations at the start of a task and the ability of HER to generalize and learn new tasks through hindsight relabeling. In a goal-reaching task, the data distribution includes states, actions, and attempted goals. HER improves the data distribution by replacing the initially desired goals with the actually achieved goals since a robot’s failure to achieve a desired goal is still a success in terms of achieving the goal it actually reached. By optimizing this non-parametric data distribution, GCIL can improve the efficiency and effectiveness of imitation learning.

### 4.2. Curriculum Learning

Curriculum learning [41] is a training method in which the complexity of the data samples used increases gradually over time. The original formulation of curriculum learning was based on the idea that it mirrors the natural way in which people learn. While it may seem that curriculum learning is solely about increasing the complexity of the training experience, it is also about leveraging the knowledge gained from simpler tasks to reduce the exploration needed in more complex tasks through generalization.

Teacher–student curriculum learning (TSCL) [42] is a method for automating the process of curriculum learning in reinforcement learning. It involves using two RL agents: a student and a teacher. The student is responsible for learning and completing tasks, while the teacher is responsible for selecting appropriate sub-tasks to help the student learn more effectively. By gradually increasing the complexity of the tasks presented to the student, the teacher helps the student learn more efficiently and effectively. TSCL is a useful approach for tackling difficult tasks that may not be able to be learned directly by breaking them down into smaller sub-tasks that can be learned more easily.

Differently from the teacher–student framework, in asymmetric self-play [43], the two agents can perform very different tasks. The two agents Alice and Bob both train on the main task directly. Alice’s role is to propose challenging goals for Bob to achieve, while Bob’s role is to try to reach those goals. This interaction between Alice and Bob creates an automated curriculum of progressively more challenging tasks, and, because Alice has already completed the task before presenting it to Bob, it is guaranteed to be achievable. Asymmetric self-play is a method for goal discovery in which the two agents work together to find and achieve new goals within the main task.

### 4.3. Hierarchical Reinforcement Learning

Hierarchical reinforcement learning (HRL) [44] is a computational approach that allows an agent to learn how to perform tasks at different levels of abstraction. It involves multiple sub-policies working together in a hierarchical framework, rather than just one policy trying to accomplish the overall goal. This method has several benefits, such as improved exploration and sampling efficiency, and can also be used for transfer learning, where low-level policies or sub-policies can be reused for multiple tasks. For example, if an agent has learned how to make coffee, it can reuse that knowledge when learning how to make a cappuccino by separating the process into making coffee and then warming and frothing the milk. HRL can, therefore, speed up the learning process for new tasks.

## 5. Network Architecture

Neural networks are function approximators that are particularly effective for deep reinforcement learning when the state space or action space is too large to be fully known. One type of neural network commonly used in deep reinforcement learning is the multi-layer perceptron (MLP), which consists of an input layer, a hidden layer, and an output layer. Each node in the hidden and output layers is a neuron with a nonlinear activation function, except for the input nodes. MLPs are trained using the supervised learning technique of backpropagation. One drawback of MLPs is that they are fully connected, meaning that each perceptron is connected to every other perceptron. This can lead to a large number of parameters and redundant information in high dimensions, making them inefficient.

### 5.1. Convolutional Neural Network

Convolutional neural networks (CNNs) [45] are particularly useful for deep reinforcement learning tasks that involve image input, such as playing video games or navigating through a visual environment. They are designed to process data with a grid-like topology, such as an image, and can learn to recognize patterns and features within the data through the use of convolutional layers. Convolutional layers apply a set of filters to the input data, which extract important features and compress the information. These features are then passed through pooling layers, which reduce the dimensionality of the data and make the network more robust to small changes in the input. The output of the CNN can then be used as input for a value or policy function in a reinforcement learning algorithm.

### 5.2. Recurrent Neural Network

A recurrent neural network (RNN) [46] is a type of artificial neural network that has connections between units that form a directed cycle. Essentially, an RNN is a loop that updates an internal state at each step while processing a sequence of inputs. One of the main features of an RNN is the hidden state, which remembers information about the sequence and is used as part of the input computation at the next time step. This allows an RNN to process sequential data, such as time series or natural language. RNNs also have the advantage of using the same parameters for each input, reducing the complexity of the model compared to other types of neural networks.

An LSTM (long short-term memory network) [47] is a type of recurrent neural network that is designed to overcome the problem of vanishing gradients in traditional RNNs. Gradients contain important information, and, if they vanish over time, important localized information can be lost. This is an issue when using RNNs for tasks such as natural language processing, for which it is important to remember information from earlier in the sequence. LSTMs address this problem by introducing “gates” with trained parameters that control the output and forgetting of the LSTM module. This helps the LSTM remember cell states and preserve important information for long periods of time. LSTMs are a more general version of gated recurrent units (GRUs), which are another type of recurrent neural network designed to handle long-term dependencies.

### 5.3. Graph Neural Network

A graph neural network (GNN) [48] is a type of neural network that takes a graph structure as input and utilizes the structural information of the graph for learning. GNNs are particularly useful for processing data that are represented in the form of a graph, as graphs can easily represent the elements and relationships within the data, making them easy to model and structure. GNNs are typically composed of smaller neural networks that represent either a node or an edge in the graph, and these smaller networks work together by passing messages to one another. GNNs are effective at exploiting knowledge graphs, such as social networks with billions of nodes representing users, photos, videos, and edges showing their relationships. These nodes and edges can contain a lot of hidden information, which a GNN can use to predict user behavior.

There are several different architectures for graph neural networks (GNNs) that can induce different inductive biases on the problem being solved. Graph convolution networks (GCN) [49] are a way to combine the embeddings of a graph node with its neighbors in a way that learns weights through averaging. In the first layer of a GCN, a node’s embeddings are weighted and averaged with its first-degree neighbors, and, in the second layer, the output for the first layer corresponding to each node is weighted and averaged with its second-degree neighbors, and so on. GraphSAGE [50] is a representation learning technique that is suitable for dynamic graphs and is capable of predicting the embedding of a new node without requiring a re-training procedure. It does this by learning aggregator functions that can induce the embedding of a new node given its features and neighborhood. The GatedGCN [51] architecture is an anisotropic message-passing-based GNN that uses residual connections, batch normalization, and edge gates. Graph attention networks (GATs) [52] are a type of anisotropic graph convolutional network that leverage an attention mechanism to learn the relative importance of neighboring nodes. By utilizing a learned self-attention weight, GATs can measure the strengths of the connections between nodes, allowing for more effective graph convolution operations.

## 6. Deep RL for Robotic Manipulation

Robotic manipulation is a classic application area for RL. Many industries, including manufacturing, supply chain, and healthcare, benefit from robotic manipulation and can benefit from the use of RL. However, robotic manipulation presents several challenges for RL, including high dimensionality, the need to deal with real-world examples, under-modeling (models that do not capture the full dynamics of the system), and uncertainty in reward and goal specification [53]. RL provides tractable approaches to addressing these challenges in robotics, including representing the state-action space through discretization, approximating the value function, and using pre-structured policies. RL can also make use of prior knowledge, such as demonstrations and task structuring, to guide exploration. Additionally, models such as mental rehearsal can help address simulation bias and deal with real-world stochasticity and optimization efficiency with simulation samples.

In the following, we discuss sim-to-real, reward engineering, value-based learning, and policy-based learning for robotic manipulation.

### 6.1. Sim-to-Real

Training a robot in simulation is often easier than training it in the real world, as RL algorithms often require a large number of samples and exploration can be risky for both the robot and the environment. However, simulators may not always accurately reflect reality, making it challenging to transfer policies trained in simulation to the real world, a process known as sim-to-real transfer. Table 4 show the list of research papers about sim-to-real implementation relative to the RL algorithms and learning techniques that they used. Peng et al. [54] proposed using dynamics randomization to train recurrent policies in simulation and deploy them directly on a physical robot, achieving good performance on an object-pushing task without calibration. However, this approach does not consider visual observations. OpenAI [55] has suggested using proximal policy optimization (PPO) with LSTMs to learn the dexterity of in-hand manipulation for a physical Shadow Dexterous Hand, using the learned policy to perform object reorientation and transferring the policy directly to the physical hand. This work shares code with OpenAI Five, a system used to play the game Dota 2.

Rusu et al. [68] proposed using progressive networks [91] to address the sim-to-real transfer problem. Progressive networks incorporate lateral connections to link the layers of previously trained network columns with each new column. This approach facilitates transfer learning, domain adaptation, and compositionality. In such networks, columns can have different capacities and structures, allowing the column intended for simulation to have greater capacity than the one designed for reality. The latter can be initialized from the former, enabling exploration and rapid learning from limited real data. Rusu et al. [68] utilized the MuJoCo physics simulator to train the first column for a reaching task with a simulated Jaco robot. They then used RGB images from a real Jaco robot to train the second column. To handle dynamic tasks, such as dynamic targets, the authors suggested incorporating a third column that utilizes proprioception features, including joint angles and velocities for the arms and fingers.

Sadeghi et al. [92] introduced a convolutional recurrent neural network for teaching a robot arm to perceive and manipulate desired objects from different viewpoints. The method utilizes previous movements as a means of selecting actions for reaching the target, rather than assuming known dynamics or requiring calibration. To learn the controller, the authors proposed using simulated demonstration trajectories in conjunction with reinforcement learning. While supervised demonstration learning often yields a policy that focuses solely on achieving the goal in the short term, RL aids in the development of a more long-term policy by assessing the action-value function to determine if the goal is attainable. Additionally, the visual layers are fine-tuned using a limited number of realistic images, which enhances the transfer performance.

Gu et al. [93] showed that a deep reinforcement learning algorithm based on off-policy training of deep Q-functions can be scaled to handle complex 3D manipulation tasks. Moreover, this approach is capable of effectively training deep neural network policies for use on physical robots. The authors utilized a normalized advantage function algorithm that enabled them to achieve training times appropriate for real-world robotic systems. Sun et al. [94] presented reinforcement learning for mobile manipulation (ReLMM), a system for autonomously learning mobile manipulation skills in the real world with minimal human intervention and without instrumentation. The authors applied stationary and autonomous curricula for grasping policy. Ding et al. [95] showed that using feedback from tactile sensor arrays located at the gripper for learning and controlling can improve grasping stability and significantly enhance the performance of robotic manipulation tasks, particularly for door-opening. This was demonstrated in both simulation and reality settings.

### 6.2. Reward Engineering

#### 6.2.1. Imitation Learning

In their discussion of reinforcement learning (RL) and the enduring issue of exploration in environments with few rewards, Nair et al. [69]. They suggest a technique that makes use of demonstrations to get around this issue and successfully learn in long-horizon, multi-step robotics tasks with continuous control, offering a speedup of an order of magnitude over RL on simulated robotics tasks and being able to solve problems that cannot be solved by RL or behavior cloning alone.

Duan et al. [96] introduced one-shot imitation learning as a supervised learning technique for scenarios with numerous tasks. The authors trained a neural network using pairs of demonstrations for a subset of tasks. Specifically, they utilized the first demonstration as input, and a state sampled from the second demonstration to predict the corresponding action. The authors incorporated soft attention to process the sequences of states and actions in a demonstration, as well as vector components for block locations (similar to those used in block stacking experiments) to enhance generalization to previously unseen conditions and tasks in the training data.

Finn et al. [97] and Yu et al. [98], independently, introduced one-shot meta-imitation learning approaches for constructing vision-based policies. These approaches fine-tune policies end-to-end from a single demonstration, using model-agnostic meta-learning (MAML) [99] to pre-train on a broad range of demonstrations from different environments. In general, learning from raw pixels necessitates a considerable amount of data. Wang et al. [100] proposed a method for robust imitation of diverse behaviors by combining the benefits of one-shot imitation learning with GAIL. The authors used a variational autoencoder on demonstration trajectories to learn semantic policy embeddings, which can be learned on a 9 DoF Jaco robot arm and result in the smooth interpolation of reaching behavior.

Riedmiller et al. [56] proposed a new learning method called scheduled auxiliary control (SAC-X) that supports the agent during learning and preserves the ability to learn from sparse rewards. The key idea behind SAC-X is to use active scheduling and execution of auxiliary policies, which allows the agent to efficiently explore the environment and excel at sparse reward RL. Andrychowicz et al. [40] studied a novel approach to dealing with sparse rewards called hindsight experience replay (HER). The idea behind HER is to replay each episode with a different goal than the one the agent was initially trying to achieve, using the final state of the episode as the new goal.

SPARTN is a technique developed by Zhou et al. [101] that enhances robot policies for eye-in-hand cameras by introducing artificial perturbations to visual displays. It does not need further professional oversight or environmental interaction because it generates corrected noise using neural radiance fields. SPARTN performs better than competing techniques in both virtual and actual grasping studies, and it does so without the need for depth sensors.

Li et al. [102] investigate how imitation learning for successful robotic manipulation is affected by demonstrator force input. Their study reveals that immersive demonstrations performed using force feedback may open up safer and speedier dexterous manipulation policies. It does this by using a feedback glove and a robot arm to depict fingertip-level and palm-level forces, respectively. As a result of force feedback, demonstrator fingertip and palm forces were dramatically decreased, resulting in less variance in forces and faster execution of recorded trajectories, according to the results. According to their study’s findings, force feedback may be essential for imitation learning to occur successfully during robotic manipulation.

Tong et al. [103] discuss the difficulty of manipulating thin items with a robot, particularly when substantial, nonlinear deformations are created. Deep neural network models are trained using a data-driven framework that combines physically realistic simulation and machine learning. The end result is a control framework that is resistant to changes in the paper’s shape and substance. Real-world tests show that, even when handling paper items of different materials and forms, the framework may significantly increase the robotic manipulation performance in comparison to natural paper folding procedures.

For visual imitation learning, Zhang et al. [104] suggest a three-phased progressive learning technique. The method is tested on a robotic pouring job and is found to offer a number of advantages over current end-to-end imitation learning systems, including a higher success rate and the capacity to generalize to other domains with less human demonstration data. The proposed approach, in the authors’ opinion, may be extensively applied to many commercial or domestic robotic manipulation applications including deep imitation learning.

Yi et al. [105] describe an autonomous grasping strategy for complex-shaped items utilizing a high-DoF robotic manipulation system made up of a four-fingered robotic hand with 16 DoFs and a 7 DoF manipulator. The system gathers data on human demonstrations using a virtual reality controller equipped with 6D position tracking and individual capacitive finger sensors. It then uses a ResNet, K-means clustering, and a point-set registration method to infer the object’s gripping stance. Five items are used to evaluate the system, and the results look good.

For robot manipulation, Wang et al. [106] provide an adaptive imitation architecture that lowers the number of human demonstrations needed to learn new tasks. In order to learn a specific class of complicated contact-rich insertion tasks based on the trajectory profile of a single task instance belonging to the task class, the system uses dynamic movement primitives in a hybrid trajectory and force learning framework. The method has been demonstrated to be more generalizable in both simulation and actual hardware environments, safer, and more sample-efficient.

For the purpose of downstream policy learning in robotic manipulation tasks, Von Hartz et al. [107] describe a technique for learning visual keypoints via dense correspondence. The method uses raw camera observations to learn picture keypoints, making policy learning more effective while addressing the issue of computationally expensive and data-intensive policy learning. The method’s adaptability and efficiency are demonstrated through evaluations of a variety of manipulation tasks and comparisons to other visual representation learning methodologies.

#### 6.2.2. Behavior Cloning

Plappert et al. [70] developed a single, goal-conditioned policy capable of solving various robotic manipulation tasks, including those with novel goals and objects. Asymmetric self-play was utilized for goal discovery, in which two agents, Alice and Bob, engaged in a game where Alice suggested difficult goals and Bob attempted to accomplish them. This technique enabled the identification of highly diverse and intricate goals without any human input.

In robotic learning challenges, Zhou et al. [108] investigate the potential for a third-person visual imitation of manipulation trajectories that is proven by embodiments that are distinct from the mimicking agent but lack access to actions. The authors provide a technique for developing manipulator-independent representations (MIR) that are ideal for cross-embodiment visual imitation with RL and are primarily concerned with the change in the environment. Cross-domain alignment, temporal smoothness, and actionability are attributes of the MIRs. With complicated robot control, the suggested technology enables agents to mimic motions from a range of embodiments with notable visual and dynamical variances, including a simulation-to-reality gap.

In order to increase the effectiveness of robotic manipulation task learning, Jung et al. [109] present a unique hybrid imitation learning (HIL) framework that combines behavior cloning (BC) and state cloning (SC) methodologies in a mutually beneficial way. The HIL framework provides stochastic state recovery to guarantee stable learning of policy networks, pre-trained dynamics networks to boost SC efficiency, and an adaptive loss mixing mechanism to efficiently blend BC and SC losses. The HIL framework demonstrated a roughly 2.6 times greater performance improvement than pure BC and a roughly four times faster training time than pure SC imitation learning in studies involving complex robotic manipulation tasks. It was superior to the BC+RL hybrid learning approach as well.

A new balancing controller for bipedal robots based on a behavior cloning model is presented by Bong et al. [110]. The controller first predicts the wrench needed to keep the bipedal robot balanced using two deep neural networks trained on data from human-operated balancing devices and then utilizes robot dynamics to compute joint torques for both legs. The created controller showed an improved performance in comparison to traditional techniques in terms of resistance to balance loss during simulation and practical tests on a bipedal lower-body robotic system. The created controller produces a better balancing motion for the robot, making it appropriate for usage in a variety of human-scale scenarios.

An imitation learning method that takes into account the diversity in expert presentations is presented by Shafiullah et al. [111]. In particular, the work suggests a novel model, dubbed the Behavior Transformer, that extracts k modes from the noisy expert demonstrations using k-means clustering. The transformer-based approach’s capacity to forecast complicated multi-modal distributions is utilized by the behavior transformer. Their study evaluates the suggested model’s performance in a number of tasks, such as Franka Kitchen, Block Pushing, and CARLA.

Piche et al. [112] suggest combining supervised learning and offline reinforcement learning (RL) to teach robotic abilities using a dataset gathered by people with varying levels of experience. The suggested technique makes use of return data and implicit models to develop robotic abilities through behavior cloning (BC). The work demonstrates that when learning robotic abilities from fixed datasets, implicit models can perform as well as or better than explicit methods. Their study also shows that the suggested strategy works well for tasks requiring high-dimensional manipulation and movement. The article provides a unified framework by demonstrating the tight connections between the suggested implicit technique and other well-known RL via supervised learning methods.

Transformers may be used in robotic manipulation jobs, which frequently need costly and restricted data, according to Shridhar et al. [113]. The authors suggest PerAct, a language-conditioned behavior-cloning agent that outputs discretized actions after using a perceiver transformer to encode language objectives and RGB-D voxel observations. PerAct offers a powerful structural prior for quickly learning 6 DoF actions since it operates on voxelized 3D observations and actions. In just a few demos per task, the authors show how PerAct can teach a single multi-task transformer to perform 18 RLBench tests and seven real-world tasks. PerAct performs better than 3D ConvNet baselines and other image-to-action agents, according to the results, for a variety of tabletop activities.

When learning from expert datasets, Wang et al. [114] use conventional behavioral cloning to outperform cutting-edge offline RL algorithms. By filtering the expert data and performing BC on the subset, a semi-supervised classification strategy is employed to enhance the outcomes while learning from mixed datasets. To make use of the environment’s geometric symmetry, simple data augmentation is also performed. The BC policies that were submitted outperformed the mean return of the corresponding raw datasets, and the policies that were trained on the filtered mixed datasets almost equaled the results of the policies that were trained on the expert datasets.

#### 6.2.3. Hierarchical RL

Finn et al. [115] investigated inverse reinforcement learning, or inverse optimal control, for control applications. The authors suggested employing nonlinear cost functions, such as neural networks, to introduce structure to the cost via informative features and effective regularization. Additionally, the authors proposed approximating MaxEnt, as proposed by Ziebart and colleagues [116], using samples for learning in high-dimensional continuous environments where the dynamics are unknown.

Li et al. [117] suggest utilizing reinforcement learning from demonstrations and temporal logic to handle the problem of learning tasks with complicated temporal structures and lengthy horizons (TL). Based on the TL job specification, the technique creates intrinsic incentives, creating a policy with an understandable and hierarchical structure. The method is tested on a variety of robotic manipulation tasks, showing that it can handle challenging situations and outperform baselines.

For the autonomous learning and enhancement of numerous gripping methods, Osa et al. [118] suggest a hierarchical policy search strategy. The technique makes use of human examples to establish the grasping strategies before automatically compiling a database of grasping actions and object point clouds to describe the grasp location and policy as a bandit issue. The framework uses reinforcement learning to grasp items that are hard and malleable and exhibits great accuracy while grabbing objects that were not before seen. The suggested method solves the problem of building a comprehensive training dataset and enables a robotic system to learn and enhance its grasping technique on its own.

To tackle problems with scarce rewards, Zhang et al. [119] suggest using a hierarchical reinforcement learning system that can learn choices independently of the task. HIDIO promotes option learning at a lower level that is independent of the job at hand, in contrast to other systems that design low-level tasks or pre-define rules. These choices are discovered using an inherent entropy reduction objective that is dependent on the sub-trajectories of the options. In studies on robotic manipulation and navigation tasks, HIDIO outperforms two cutting-edge hierarchical RL approaches and ordinary RL baselines in terms of success rates and sampling efficiency.

#### 6.2.4. Generative Adversarial Imitation Learning (GAIL)

In order to make the computation fully differentiable in policy imitation problems, Baram et al. [120] offer a novel approach called model-based generative adversarial imitation learning (MGAIL). As a result, policies may be trained utilizing the precise gradient of the discriminator, leading to more competent policies with relatively fewer expert samples and environmental interactions. The technique outperforms the most recent algorithms in tests conducted on both discrete and continuous action domains.

Merel et al. [121] suggest utilizing generative adversarial imitation learning to train controllers for high-dimensional humanoid bodies. The technique enables training of general neural network policies to generate movement patterns similar to those of humans from a small number of demonstrations, without access to actions, and even when the demonstrations originate from a body with diverse and unidentified physical attributes. Motion capture data are used to create sub-skill rules, which may then be utilized to perform tasks when commanded by a higher-level controller. Compared to pure reinforcement learning with straightforward reward functions, the method produces movement behaviors that are more varied and human-like.

In order to robustly learn desirable policies even from subpar demonstrations, Tsurumine et al. [122] propose goal-aware generative adversarial imitation learning (GA-GAIL), which uses a second discriminator to distinguish the goal state in parallel with the first discriminator that indicates the demonstration data. To accomplish stable policy learning from two discriminators, GA-GAIL uses the entropy-maximizing deep P-network (EDPN) as a generator. The suggested technique learned cloth manipulation policies without a task-specific reward function design when it was successfully applied to two real robotic fabric manipulation challenges.

According to Zolna et al. [123], adversarial imitation tasks are performed poorly because the discriminative networks have the propensity to focus on qualities that are unrelated to the job at hand. They suggest task-relevant adversarial imitation learning (TRAIL), which uses limited discriminator optimization to learn instructive rewards, to overcome this. TRAIL performs better than traditional GAIL at difficult pixel manipulation tasks by mimicking human operators without using any task incentives. The findings further demonstrate that TRAIL significantly outperforms baseline imitation agents with comparable performances, including those taught by behavior cloning and standard GAIL.

#### 6.2.5. Curriculum Learning

Abstract demonstrations and adaptive exploration are two deep reinforcement learning (DRL) exploration techniques that Yang et al. [124] offer. These two elements are inspired by human experiences. A2 breaks down a challenging activity into manageable parts and suggests the right learning sequences for each part. Throughout training, the agent adaptively explores its surroundings, responding more deterministically for sub-tasks that it has learned and more stochastically for those that it has not. In grid-world and robotic manipulation tasks, A2 enables common DRL algorithms (DQN, DDPG, and SAC) to learn more effectively and steadily.

In order to overcome the difficulty of planning and building a bridge without a blueprint, Li et al. [125] suggest a bi-level strategy. The system uses deep reinforcement learning and curriculum learning to acquire a bridge blueprint policy in a physical simulator. In order to provide real-robot motion control, a motion-planning-based policy is built for low-level control. This policy may be immediately merged with a learned blueprint policy for actual bridge construction without tuning. It is shown that the bi-level robot system is capable of using blocks to build a variety of bridges with various architectural styles.

#### 6.2.6. Transfer Learning

The KOVIS approach, developed by Puang et al. [126] employs an eye-in-hand stereo camera system and a deep neural network that has been trained only in a simulated environment to carry out delicate robotic manipulation tasks. Two networks, a keypoint network and a visual servoing network, that were trained end-to-end and under self-supervision make up KOVIS. The suggested technique demonstrates its efficacy in both simulated and real-world situations by achieving zero-shot sim-to-real transfer to robotic manipulation tasks such as grabbing, peg-in-hole insertion with 4 mm clearance, and M13 screw insertion.

Using inputs from a single camera, Yuan et al. [127] provide a sim-to-real learning approach for vision-based assembly jobs that addresses safety constraints and sample efficiency problems in training robots in the real world. The system contains a force control transfer mechanism to close the reality gap as well as a domain adaptation technique based on cycle-consistent generative adversarial networks (CycleGAN). The suggested framework may be effectively applied to a genuine peg-in-hole arrangement after being taught in a simulated environment.

A new technique for predicting domain randomization distributions for secure sim-to-real transfer of reinforcement learning policies in robotic manipulation is introduced by Tiboni et al. [128] as DROPO. Unlike earlier research, DROPO only needs a small, offline dataset of trajectory data that has been acquired in advance, and it directly represents parameter uncertainty using a likelihood-based method. The article shows how DROPO can recover dynamic parameter distributions during simulation and identify a distribution that can account for unmodeled phenomena. Two zero-shot sim-to-real transfer scenarios were used to test the method, which demonstrates effective domain transfer and enhanced performance over earlier approaches.

The Kalman Randomized-to-Canonical Model, a zero-shot sim-to-real transferable visual model predictive control (MPC) technique, is presented by Yamanokuchi et al. [129]. The suggested system utilizes the KRC model to extract intrinsic characteristics and dynamics that are task-relevant from randomized pictures. Via a block-mating task in simulation and a valve-rotation task in both the real world and simulation, the effectiveness of the technique is assessed. The findings demonstrate that KRC-MPC may be utilized in a zero-shot way across a range of real domains and activities without the need for any actual data collection.

**Figure 9:**
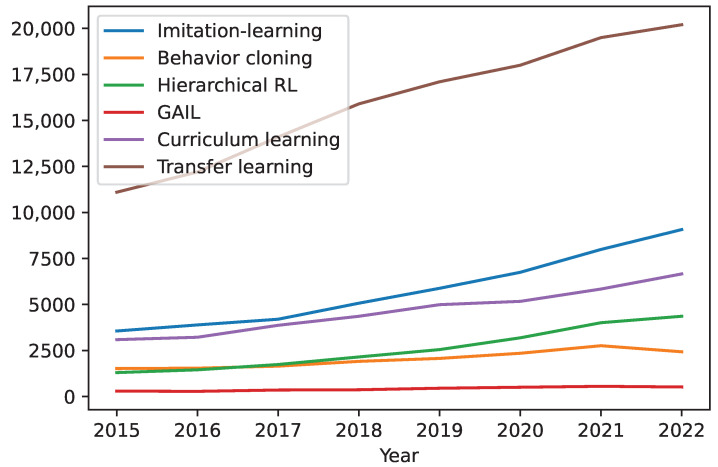
The trend of published papers using different reward engineering in robotic manipulation.

For robot manipulators, there are multiple reward engineering strategies available. Figure 9 shows the trend for reward engineering used in robotic manipulation from 2015 to 2022.

### 6.3. RL Techniques

#### 6.3.1. Q-Learning

The difficulty of modifying robot learning systems to new surroundings, objects, and perceptions is covered by Julian et al. [130]. Their paper offers a framework for fine-tuning previously acquired behaviors through Q-learning, which promotes continual adaptation. Their method has been demonstrated to be successful in a scenario of continuous learning and results in significant performance increases with little data. Experiments on simulated manipulation tasks and a genuine robotic grasping system that has been pre-trained on 580,000 grasps provide support for the findings.

#### 6.3.2. Deep Q-Network

Due to the number of samples necessary for precise estimations, learning continuous control in high-dimensional, sparse reward environments, such as robotic manipulation, is a difficult challenge. For these tasks, Rammohan et al. [131] recommend value-based reinforcement learning, more especially RBF-DQN. For robotic manipulation tasks, they show that RBF-DQN converges more quickly than existing state-of-the-art algorithms, such as TD3, SAC, and PPO, and that RBF-DQN is also amenable to improvement methods, such as HER and PER. The authors contend that compared to policy gradient approaches, value-based systems may be more susceptible to data augmentation and replay buffer sampling strategies.

The use of equivariant neural networks in Q-learning and actor–critic reinforcement learning is investigated by Wang et al. [132]. Equivariant models are suited to robotic manipulation issues because they enforce symmetry in their convolutional layers and can considerably increase sampling efficiency when learning an equivariant or invariant function. The authors suggest equivariant variations of the DQN and SAC algorithms that take advantage of this structure and show through tests that they can be more sample-efficient than rival algorithms on certain robotic manipulation challenges.

Manipulation question answering (MQA), a novel task proposed by Deng et al. [133], requires the robot to manipulate the surroundings in order to answer a question. The suggested system consists of a QA module and a manipulation module that generates manipulation actions for the robot to interact with the environment using a deep Q-network (DQN) model. In order to verify the efficacy of the suggested framework, the authors additionally create a fresh dataset in a simulation environment that includes a range of object models, situations, and related question–answer pairs. They also carry out comprehensive tests.

A deep reinforcement learning (RL) framework for pick-and-place tasks in crowded industrial contexts is proposed by Imtiaz et al. [134]. Using a deep Q-network (DQN) made up of three fully convolutional networks (FCN) based on the DenseNet-121 architecture, the issue is handled as a Markov decision process (MDP). After each forward pass, prizes are distributed based on the affordance maps that the FCNs produce for each action. The suggested framework outperforms a baseline deep learning technique and a ResNet architecture-based approach, according to experiments, and achieves promising results in a variety of challenging cases.

A pushing policy is suggested by Sarantopoulos et al. [135] to remove a known target object from a mass of unrelated objects in a crowded environment. The method learns the best push policies using deep Q-learning (DQN), and a split DQN is suggested to increase the learning rate and modularity. The comprehensive feature selection ensures that the learned rules perform well in both simulated and actual contexts. The algorithm’s modularity enables the insertion of additional primitives without having to completely retrain the model.

#### 6.3.3. Proximal Policy Optimization

Chen et al. [59] aimed to address multiple robotic manipulation tasks, such as grasping, button-pushing, and door-opening, using reinforcement learning (RL), state representation learning (SRL), and imitation learning. To achieve this, the authors built simulated environments in PyBullet and explored three different learning-style methods using wrapped PPO and DQN algorithms from the OpenAI baseline. These methods were successfully applied to solve diverse missions in the self-constructed environments.

By storing recovery actions in a separate safety buffer and utilizing k-means clustering to choose the optimum recovery action when coming across comparable situations, Hsu et al. [136] provide a method for assuring safety in deep reinforcement learning (RL). Six robotic control tasks including navigation and manipulation are used to assess the proposed safety-aware RL algorithm. The results demonstrate that it outperforms numerous baselines in both discrete and continuous control problems, increasing safety throughout both the training and testing phases.

Iriondo et al. [137] provide a technique for picking items up in a mobile manipulator in unstructured regions. For such activities, conventional path planning and control become difficult. In this work, a controller for the robot base is trained using a deep reinforcement learning (DRL) technique. This controller directs the platform to a location where the arm may plan a route up to the item. The effectiveness of the DRL technique is assessed using two DRL algorithms (DDPG and PPO), which are contrasted, and a specific robotic job.

#### 6.3.4. Trust Region Policy Optimization

A technique for training a neural network policy in simulation and applying it to a cutting-edge legged system, such as the ANYmal robot, is presented by Hwangbo et al. [138]. The method provides quick, automated, and economical data-generating processes. The quadrupedal robot demonstrates locomotion abilities that are beyond those of other techniques: ANYmal is able to correctly and effectively obey high-level body velocity commands, run faster than before, and recover from falls even in complicated configurations.

Clegg et al. [139] investigate the application of reinforcement learning to create robotic dressing aides that can predict human movements. The robot offers a client an open sleeve of a medical gown, and the individual puts their arm into the sleeve, according to the researchers’ models of human behavior during dressing assistance. The system develops a model of human behavior using the TRPO algorithm that can effectively train for three distinct robot-assisted dressing procedures and can insert the arm into the sleeve. The purpose of the project is to simulate how individuals may aid with dressing.

#### 6.3.5. Deep Deterministic Policy Gradient

Vecerik et al. [57] developed a model-free reinforcement learning approach for real robotics with sparse rewards by using demonstrations and extending the deep deterministic policy gradient (DDPG) algorithm. Both demonstrations and actual interactions were used to populate a replay buffer, and a prioritized replay mechanism [140] automatically adjusted the sampling ratio between demonstrations and transitions. Kilinc et al. [58] proposed a novel framework for robotic manipulation that does not rely on human demonstrations. They framed every robotic manipulation task as a locomotion task from the perspective of the manipulated object and used a physics simulator to obtain an object locomotion policy. This policy was then used to generate simulated locomotion demonstration rewards (SLDRs), which enable the learning of the robot manipulation policy. Yang et al. [62] developed a Pybullet engine re-implementation of the OpenAI multi-goal robotic manipulation environment with additional APIs for joint control, customizable camera goals and image observations, and on-hand camera access. The authors also created a series of challenging robotic manipulation tasks with sparse rewards, long horizons, and multi-step and multi-goal objectives to inspire new goal-conditioned reinforcement learning algorithms. Vulin et al. [63] tackled the challenge of exploration in deep reinforcement learning for robotic manipulation by introducing an intrinsic reward based on the sum of forces between the robot’s force sensors and manipulation objects, encouraging physical interaction. They also proposed a contact-prioritized experience replay sampling scheme that prioritizes contact-rich episodes and transitions.

Residual policy learning (RPL) [64] is a method for improving policies using model-free deep reinforcement learning. It allows the gradient to backpropagate through the value of the next state, rather than treating it as a black box. RPL has been shown to consistently and substantially improve initial controllers. DDPG, a reinforcement learning algorithm, can be made more efficient and robust for complex continuous control tasks through two extensions proposed by Popov et al. [67]: decoupling the frequency of network updates from the environment interaction and using an asynchronous version that allows data collection and network training to be distributed over multiple computers. Nair et al. [69] developed a system that combines demonstrations with reinforcement learning to solve multi-step tasks, which can be useful for tasks with sparse rewards or other exploration challenges. However, this method may not be sample efficient for more difficult tasks.

Using a TriFinger robot that won Phase 1 of the Real Robot Challenge (RRC) 2021, Wang et al. [141] present a deep reinforcement learning (DRL) approach for manipulating a cube. They then expand this approach to a more challenging manipulation task where the robot must maintain the cube in a specific orientation. The learning of the DRL agent (DDPG) in the original work was guided by a mix of goal-based sparse rewards and distance rewards with hindsight experience replay (HER), and the acquired techniques were transferred to the expanded task using a knowledge transfer (KT) approach. The longer job was learned and performed by the agent with an enhanced performance during evaluation thanks to the KT approach.

Hindsight goal ranking (HGR), a technique introduced by Luu et al. [142], prioritizes the replay experience for reinforcement learning by sampling more frequently on the states visited in an episode with a bigger temporal difference (TD) error. To increase the learning effectiveness for robotic manipulation tasks, HGR is paired with deep deterministic policy gradient (DDPG), an off-policy model-free actor–critic technique. The findings demonstrate that, across all activities, HGR considerably accelerates learning more quickly than the prior techniques.

Eppe et al. [143] provide a generic goal-masking technique that permits deep reinforcement learning curriculum learning (DRL). The strategy focuses on improving the goal sampling process to enhance the learning performance by predicting the level of goal complexity. The findings demonstrate that, for DDPG approaches, objectives with medium difficulty levels are adequate, but combining DDPG with HER benefits from a “reach for the stars” strategy in which challenging goals are selected more often. The method performs better than conventional goal sampling in a variety of robotic object manipulation issues.

Sehgal et al. [144] suggest using a genetic algorithm (GA) to determine the ideal parameters for the deep deterministic policy gradient (DDPG) algorithm in combination with hindsight experience replay in order to optimize the learning process of reinforcement learning (RL) agents in robotic manipulation tasks (HER). Five robotic manipulation tasks, including fetch-reach, slide, push, pick and place, and door-opening, are subject to the method’s application. According to the experimental findings, the suggested solution outperforms the original algorithm in terms of the learning performance both quickly and effectively. For six robotic manipulation tasks, FetchReach, FetchSlide, FetchPickAndPlace, DoorOpening, and AuboReach [145], they also suggest using a genetic algorithm (GA) technique to fine-tune the hyperparameters of deep deterministic policy gradient (DDPG) along with Hindsight Experience Replay (HER). The performance of the suggested GA+DDPG+HER approach is significantly higher than that of the current methods, resulting in a reduction in learning time. The experimental findings demonstrated the usefulness of the suggested approach in accelerating reinforcement learning agents’ learning rates.

#### 6.3.6. Twin Delayed Deep Deterministic Policy Gradients

The approach put out by Nair et al. [146] will allow an autonomous agent to develop a wide range of general-purpose skill repertoires for attaining user-specified goals during testing. The approach combines unsupervised representation learning and reinforcement learning of goal-conditioned policies. In order to envision objectives and try to attain them, the agent goes through a self-supervised practice phase. To increase sample effectiveness, a retroactive goal relabeling technique is suggested. A visual representation is learned to sample objectives, manipulate sensory inputs, and calculate a reward signal. The algorithm outperforms earlier methods and is effective enough to work on unprocessed picture observations and objectives for practical robotic systems.

In order to reduce tracking inaccuracy and optimize the communication burden between a physical device and its digital model in the metaverse, Meng et al. [147] offer a co-design framework. The system uses the restricted deep reinforcement learning (DRL) algorithm KC-TD3, which makes use of expert knowledge to maximize the sampling rate and prediction horizon. In comparison to previous methods, the framework achieves a superior trade-off between the average MSE and the average communication load on a prototype consisting of a robotic arm and its digital model. With the use of expert knowledge, the suggested method also achieves a higher convergence time, stability, and final policy performance.

#### 6.3.7. Soft Actor–Critic

Zhang et al. [60] propose a novel intrinsic regularization method for training a policy for bimanual manipulation tasks involving multiple objects. The goal is for the agents to learn how to allocate a workload and avoid domination and conflict. They use a self-attention architecture to combine the embedded representations of the agents and objects and compute an intrinsic loss based on attention probabilities to encourage the agents to focus on different sub-tasks. Yamada et al. [61] present a flexible framework that combines motion planning and reinforcement learning for efficient learning of continuous robot control in cluttered environments. The framework combines a model-free RL approach with a sampling-based motion planner that requires minimal task-specific knowledge. The RL policy learns when to use the motion planner and when to take a single-step action through reward maximization.

Zhan et al. [71] present a framework that combines demonstrations, unsupervised learning, and RL to efficiently learn complex tasks in the real world using only image input. They are able to solve a variety of tasks using the same hyperparameters and with only sparse rewards. Using only 10 demonstrations, their method allows a single robotic arm to learn manipulation policies from pixels. The low amount of supervision required makes this approach promising for efficiently applying RL to real robots.

Reinforcement learning is used by Li et al. [148] to solve the problem of automating instrument delivery in vascular intervention surgery. Existing reinforcement learning techniques are constrained by the non-linear connection between manipulation commands and instrument movements in actual vascular robotic systems. The research suggests DSAC-AE, a discrete soft actor–critic algorithm enhanced with an additional reconstruction job, as a solution to this issue. The algorithm is used in a robot-assisted clinical scenario with dispersed sample collection and parameter updating, proving its capacity to pick up manipulation techniques for vascular robotic systems after 50k sampling steps in less than 15 h.

The application of equivariant neural network models in on-robot policy learning for robotic manipulation tasks is investigated by Wang et al. [149]. Without using a model, simulator, or offline dataset, equivariant SAC is used to learn policies solely on a physical robotic system. The results demonstrate the capability to fully learn non-trivial manipulation tasks through on-robot encounters in less than an hour or two of wall clock time after exploring a number of iterations of the method.

To improve the robustness of deep reinforcement learning policies in robotic manipulation tasks, Jian et al. [150] present an adversarial skill learning method based on soft actor–critic (SAC). It is determined that the algorithm is resilient to both internal and external disturbances in both simulation and real-world contexts.

**Figure 10:**
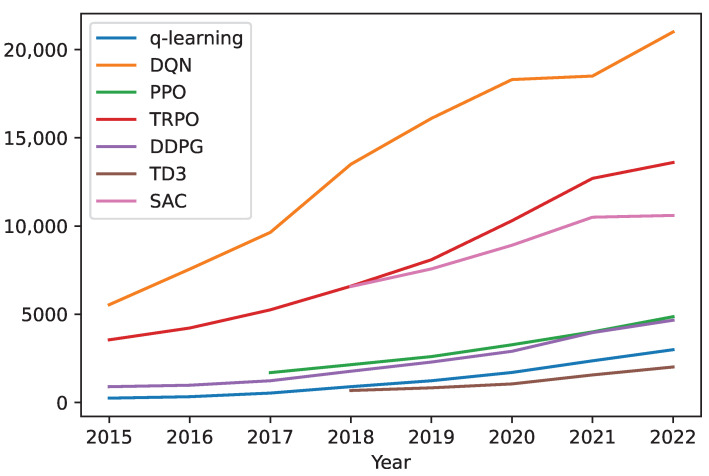
The trend of published papers using different RL algorithms in robotic manipulation.

There are lots of RL techniques for robot manipulators to apply. Figure 10 categorizes the research papers by using different RL algorithms in robotic manipulation from 2015 to 2022.

#### 6.3.8. GNN

Graph neural networks (GNNs) can be used as the network architecture for reinforcement learning problems in relational environments. Table 5 features a list of papers about GNN implementation relative to RL algorithms and learning techniques. Janisch et al. [151] proposed a deep RL framework based on GNNs and auto-regressive policy decomposition that is well-suited to these types of problems. They demonstrate that their approach, which uses GNNs, can solve problems and generalize to different sizes without any prior knowledge.

To address the issue of limited generalization in current DRL-based networking solutions, Almasan et al. [152] present a deep reinforcement learning (DRL) agent including graph neural networks (GNN). The proposed GNN-based DRL agent can learn and generalize over diverse network topologies since GNNs are built to generalize over networks of various sizes and architectures. The agent outperforms cutting-edge methods in topologies not seen during training in a routing optimization use case in optical networks.

Richard Li et al. [66] present a reinforcement learning system that can stack six blocks without any demonstrations or task-specific assumptions. They use the soft actor–critic (SAC) algorithm as their base learning algorithm because it is more robust to hyperparameter choices and random seeds than other off-policy learners such as deep deterministic policy gradient (DDPG). They find that training a policy represented by an attention-based graph neural network (GNN) enables successful curriculum learning in multi-object manipulation tasks.

Yunfei Li et al. [125] study a challenging assembly task in which a robot arm must build a feasible bridge based on a given design. The authors divide the problem into two steps. First, they use an attention-based neural network as a “blueprint policy” to assemble the bridge in a simulation environment. Then, they implement a motion-planning-based policy for real-robot motion control.

Lin et al. [153] present a method for robot manipulation using graph neural networks (GNNs). The authors propose a GNN-based approach for solving the object grasping problem, in which the robot must select a suitable grasping pose for a given object. The approach combines a graph representation of the object with a GNN to learn a grasp quality function, which predicts the success of a grasp based on the object’s geometry and the robot’s kinematics. The authors evaluate their method on a dataset of synthetic objects and a real-world grasping task and demonstrate that it outperforms previous approaches in terms of both efficiency and interpretability.

According to the review above, it appears that deep reinforcement learning has been effectively used for a range of robotic manipulation tasks, such as grasping, pushing, and door-opening. For these tasks, a variety of strategies have been put forth to increase the sample efficiency of RL, including the use of demonstrations, supplemental incentives, and experience replay. It has also been demonstrated that other techniques, such as model-free approaches, residual learning, and asymmetric self-play, are useful for robotic manipulation. Overall, it seems that there is still a lot of opportunity for future study in this field, especially in terms of figuring out how to learn complicated manipulation tasks with scarce incentives more effectively and in terms of applying learned rules from a simulation to the real world.

### 6.4. Future Directions

In the field of robotic manipulation, there are numerous potential future avenues for deep reinforcement learning algorithms:

Improving sample efficiency: Currently, most deep reinforcement learning algorithms for robotic manipulation require a large number of samples to learn a task. One future direction is to develop algorithms that can learn from fewer samples or to find ways to reuse the samples more efficiently.

Transfer learning: The development of algorithms that can transfer information from one job to another or from one robot to another is another path for transfer learning. Robots would be able to learn new tasks more quickly and simply as a result of this.

Real-time control: Many current deep reinforcement learning algorithms are too slow for real-time control of robots. Improving the speed of these algorithms would allow them to be used in a wider range of applications.

Safe exploration: Robots must be capable of safely exploring their surroundings in order to avoid harming themselves or their surroundings, as well as to learn from their mistakes. Future research should focus on creating algorithms that really can balance the demand for exploration with the necessity for safety.

Integrating with other learning paradigms: Another approach is to develop algorithms that integrate supervised and unsupervised learning, including reinforcement learning, with other learning paradigms. This could make it possible for robots to learn more difficult jobs that need a mix of learning methods.

## 7. Conclusions

In this survey, we have provided an overview of deep reinforcement learning algorithms for robotic manipulation. We have discussed the various approaches that have been taken to address the challenges of learning manipulation tasks, including sim-to-real, reward engineering, value-based, and policy-based approaches. We have also highlighted the key challenges that remain in this field, including improving sample efficiency, developing transfer learning capabilities, achieving real-time control, enabling safe exploration, and integrating with other learning paradigms. One of the key strengths of the survey is its comprehensive coverage of the current state-of-the-art in DRL and exploring a wide range of techniques and applications for robotic manipulation. The survey does not, however, go into great depth on all strategies due to space considerations. Nevertheless, the survey can greatly benefit researchers and practitioners in the field of robotics and reinforcement learning by providing insights into the advantages and limitations of various algorithms and guiding the development of new approaches. Overall, this survey serves as a valuable resource for understanding the current landscape of deep reinforcement learning in robotic manipulation and can inspire further research to advance the field.

Future research in this field should concentrate more on overcoming these difficulties and figuring out how to improve the performance and efficiency of deep reinforcement learning algorithms for robotic manipulation tasks. By achieving this, we can get one step closer to developing intelligent robots that can adjust to different surroundings and learn novel abilities on their own. It is likely that as the area of RL for robotic manipulations develops, we will witness the creation of increasingly more sophisticated algorithms and methodologies that will allow robots to perform increasingly difficult manipulation tasks.

## Figures and Tables

**Table 1:** An overview of the specified search criteria.

Criteria	Description
Keywords	“reinforcement learning” AND “robotic manipulation” AND “manipulation tasks” AND “control policies”
Search engine	Google Scholar, IEEE Xplore, or ArXiv
Time period	Between 2015 and the present
Publication type	Academic conference paper and journal articles
Relevance	Exclude studies that are not appropriate for the scope of the review
Outdated	Exclude old papers that are no longer relevant to the current state of the field
Quality	Exclude poorly written or methodologically flawed papers

**Table 2:** Categories of different RL algorithms.

Methods	Value-Based	Policy-Based	Actor–Critic
On-Policy	Monte Carlo Learning/ SARSA	REINFORCE(PG)	A2C/ A3C/ TRPO/ PPO
Off-Policy	Q-learning/ DQN Double/ Dueling		DDPG/ TD3/ SAC

**Table 3:** The strengths and limitations of different RL algorithms.

RL Algorithm	Strengths	Limitations
Q-Learning	Straightforward and easy to use, capable of handling discrete state and action spaces, eventually converges to the best possible policy	Suffers from slow convergence, difficulty using in continuous action spaces
SARSA	Manages stochastic environments and policies	Slow convergence and difficulty using in continuous action areas
Deep Q-Networks (DQN)	Handle high-dimensional state spaces, directly learns from sensory data	Difficulty using in continuous action spaces, overestimation of Q-values
Policy Gradient	Handles continuous action spaces, learns stochastic policies, optimizes non-differentiable objective functions	High variance in gradients, sensitive to hyperparameters
Actor–Critic	Combines the advantages of policy gradient and value-based approaches, handles continuous and discrete action spaces, concurrently updates policy and value function	Difficult to balance exploration and exploitation, high variance in gradients
DDPG	Handles continuous action spaces and high-dimensional state spaces, learns deterministic policies,	Instability, overestimation bias
TD3	Same as DDPG and solves overestimation bias	Instability, careful tuning of hyperparameters
SAC	Same as TD3 and optimizes entropy-regularized objectives	Computationally expensive, careful tuning of hyperparameters

**Table 4:** A list of papers about sim-to-real implementation relative to RL Algorithms and learning techniques.

Methods	RL Algorithms	Learning Techniques
Learning by playing and solving sparse reward tasks from scratch [56]	Q-learning and DDPG	Scheduled auxiliary control and hierarchical RL
Leveraging demonstrations for deep reinforcement learning on robotics problems with sparse rewards [57]	DDPG	Demonstration and reward shaping
Reinforcement learning for robotic manipulation using simulated locomotion demonstrations [58]	DDPG and HER	Simulated demonstrations and behavior cloning
Robotic manipulation with reinforcement learning, state representation learning, and imitation learning [59]	PPO and DQN	State representation learning (SRL)
Disentangled attention intrinsic regularization for safe and efficient bimanual manipulation [60]	SAC	Attention mechanism
Motion planner augmented reinforcement learning for robot manipulation in obstructed environments [61]	SAC	Motion planning augment action space re-scaling
Open-source multi-goal reinforcement learning environment for robotic manipulation [62]	DDPG and HER	Human-prior-based curriculum learning
Improved learning of robot manipulation tasks via tactile intrinsic motivation [63]	DDPG and CPER	Intrinsic motivation and tactile feedback
Residual policy learning [64]	DDPG and HER	Residual policy learning
Learning to control a low-cost manipulator using data-efficient reinforcement learning [65]	Model-based approach	Transfer learning
Towards practical multi-object manipulation using relational reinforcement learning [66]	SAC and HER	Sequential curriculum learning
Data-efficient deep reinforcement learning for dexterous manipulation [67]	DDPG and A3C	Reward shaping
Sim-to-real robot learning from pixels with progressive nets [68]	A3C	Transfer learning
Overcoming exploration in reinforcement learning with demonstrations [69]	DDPG and HER	Imitation learning
Asymmetric self-play for automatic goal discovery in robotic manipulation [70]	PPO	Asymmetric self-play
A framework for efficient robotic manipulation [71]	SAC	Imitation learning
Robotic arm control and task training through deep reinforcement learning [72]	DQN and TRPO	Curriculum learning
Method of robot grasping based on reinforcement learning [73]	DQN	Priority experience replay
Residual learning from demonstration: adapting dmps for contact-rich manipulation [74]	PPO	Behavior cloning
Learning insertion primitives with discrete-continuous hybrid action space for robotic assembly tasks [75]	Multi-pass DQN	Transfer learning
Offline meta-reinforcement learning for industrial insertion [76]	A2C	Demonstration
Impedance control and parameter optimization of surface polishing robot based on reinforcement learning [77]	PG	Dynamic matching and linearization
Robotic architectural assembly with tactile skills: Simulation and optimization [78]	TD3	Transfer learning
Manipulation planning from demonstration via goal-conditioned prior action primitive decomposition and alignment [79]	DAPG	Imitation learning and hierarchical RL
Reinforcement learning with vision-proprioception model for robot planar pushing [80]	SAC	Variational autoencoder
Object manipulation system based on image-based reinforcement learning [81]	SAC	Transfer learning
Augmenting reinforcement learning with behavior primitives for diverse manipulation tasks [82]	MAPLE	Hierarchical RL
Evaluation of variable impedance and hybrid force/motion controllers for learning force tracking skills [83]	Model-based PILCO [84]	Hybrid force/motion control
Context meta-reinforcement learning via neuromodulation [85]	SAC	Meta-RL
Safe learning in robotics: from learning-based control to safe reinforcement learning [86]	PPO	Model predictive safety certification [87]
Prioritized hindsight with dual buffer for meta-reinforcement learning [88]	SAC	Prioritized hindsight with dual experience replay
Object detection-based one-shot imitation learning with an RGB-D camera [89]	PG	Auto-encoder and object detection network
Retinagan: an object-aware approach to sim-to-real transfer [90]	Q2-OPT	GAN and imitation learning

**Table 5:** A list of papers about GNN implementation relative to RL algorithms and learning techniques.

Reference Papers	RL Algorithms	Learning Techniques
[152]	DQN and GNN	Evaluate on OTN routing map
[125]	Blueprint policy and PPG	Curriculum learning
[151]	GNN and A2C	Behavior cloning
[153]	PPO	Imitation learning
[66]	GNN and SAC	Sequential curriculum learning
[154]	Model-based method	Imitation learning
[155]	MDP and GAT	Hierarchical imitation learning
[156]	PG and GCN	GAIL
[157]	PPO and GCN	Relational Inductive Bias

## Data Availability

Not applicable.
